# Non-traumatic complete cervical spine dislocation with severe fixed kyphosis: successful multidisciplinary approach to a challenging case

**DOI:** 10.1186/s13256-024-04446-x

**Published:** 2024-04-01

**Authors:** Camille Lecouvet, Pierre Geradon, Xavier Banse, Gauthier Rausin, Nicolas Guyot, Frederic E. Lecouvet

**Affiliations:** 1 Department of Anesthesia, Institut de Recherche Expérimentale et Clinique (IREC), Cliniques Universitaires Saint-Luc, UCLouvain, Hippocrate Avenue 10, 1200 Brussels, Belgium; 2 Department of Orthopedic Surgery, Institut de Recherche Expérimentale et Clinique (IREC), Cliniques Universitaires Saint-Luc, UCLouvain, Hippocrate Avenue 10, 1200 Brussels, Belgium; 3 Department of Medical Imaging, Institut de Recherche Expérimentale et Clinique (IREC), Cliniques Universitaires Saint-Luc, UCLouvain, Hippocrate Avenue 10, 1200 Brussels, Belgium

**Keywords:** Cervical spine, Joint dislocation, Spinal injuries, Anesthesia, MRI

## Abstract

**Background:**

To our knowledge, there is no previous report in the literature of non-traumatic neglected complete cervical spine dislocation characterized by anterior spondyloptosis of C4, extreme head drop, and irreducible cervicothoracic kyphosis.

**Case presentation:**

We report the case of a 33-year-old Caucasian man with a 17-year history of severe immune polymyositis and regular physiotherapy who presented with severe non-reducible kyphosis of the cervicothoracic junction and progressive tetraparesia for several weeks after a physiotherapy session. Radiographs, computed tomography, and magnetic resonance imaging revealed a complete dislocation at the C4–C5 level, with C4 spondyloptosis, kyphotic angulation, spinal cord compression, and severe myelopathy. Due to recent worsening of neurological symptoms, an invasive treatment strategy was indicated. The patient’s neurological status and spinal deformity greatly complicated the anesthetic and surgical management, which was planned after extensive multidisciplinary discussion and relied on close collaboration between the orthopedic surgeon and the anesthetist. Regarding anesthesia, difficult airway access was expected due to severe cervical angulation, limited mouth opening, and thyromental distance, with high risk of difficult ventilation and intubation. Patient management was further complicated by a theoretical risk of neurogenic shock, motor and sensory deterioration, instability due to position changes during surgery, and postoperative respiratory failure. Regarding surgery, a multistage approach was carefully planned. After a failed attempt at closed reduction, a three-stage surgical procedure was performed to reduce displacement and stabilize the spine, resulting in correct spinal realignment and fixation. Progressive complete neurological recovery was observed.

**Conclusion:**

This case illustrates the successful management of a critical situation based on a multidisciplinary collaboration involving radiologists, anesthesiologists, and spine surgeons.

## Background

Complete cervical spine dislocation is a serious condition resulting from a distraction–flexion injury with an evident traumatic origin in the vast majority of cases, causing damage to the stabilizing osseous and soft tissue structures of the cervical spine [1, 2]. This lesion can be axial, involving the cervicooccipital or atlantoaxial articulations, or subaxial, affecting the C2–T1 segment [3]. Motor vehicle accidents and falls are the most frequent causes, predominantly observed in young adults and elderly people, respectively [4]. Most often, a history of recent spinal trauma is present. However, post-traumatic cervical dislocation may be ignored at the early phase, and “delayed” or “neglected” presentations of dislocation have been reported [5, 6].

Beside muscular strains and ligament tears, spine dislocations may be associated with fractures of vertebral bodies or posterior arch, canal stenosis, spinal cord compression, and injury with severe neurological deficits. The diagnosis of dislocation is usually made during the early imaging workup using radiographs, computed tomography (CT), and magnetic resonance imaging (MRI), which provide information on bones, discs, posterior stabilizing structures, spinal cord compression, and possible myelopathy [7]. Complete dislocation with “locked facets” and traumatic spondyloptosis are the most severe forms of this spectrum of lesions [8]. The severity and risk of worsening neurological damage to the spinal cord mean that early and careful medical attention, as well as surgical intervention, is often required. Treatment may consist of closed reduction with cranial traction or open reduction followed by spinal fusion [9, 10].

We report the case of complete non-traumatic C4–C5 dislocation with C4 spondyloptosis in a patient with a predisposing neuromuscular disease. After an exhaustive imaging workup, a multistep surgical approach was planned. The neurologic status of the patient and severe cervicothoracic kyphosis represented a critical challenge for the anesthesiologist. As previously suggested in the literature, the importance of the multidisciplinary collaboration between clinicians, radiologists, anesthesiologists, and surgeons for the successful management of this challenging spine dislocation is underlined [11].

This publication adheres to our Institutional Review Board policies regarding confidentiality and anonymization of medical data, and signed informed consent was obtained from the patient for publication of this case report and accompanying images.

## Case presentation

### Clinical history and imaging workup

A 33-year-old Caucasian male patient presented with progressive deterioration of neurological status and development of severe head flexion on the trunk. He had a previous history of chronic severe muscle atrophy due to autoimmune myositis, which had appeared in adolescence and was responsible for mild diffuse weakness in adulthood, but with a normal neurological status and no impact on his autonomy. He also had an old history of sporadic Duane retraction syndrome that occurred at the age of 12 years and was responsible for residual oculomotor limitation. He had no other previous medical history and there was no particular medical familial history.

His symptoms had started 3 months prior to his admission after one of his regular physical therapy sessions. These initial symptoms consisted of episodes of intermittent pain, difficulty turning his head, moving his shoulders, and lifting his arms. These symptoms had been neglected until they became permanent.

For 1 week prior to admission, the patient now described feeling as if his head was “completely stuck.”

The day of admission, the clinical examination revealed severe non-reducible flexion of the head on the chest, with the chin almost in contact with the sternum, and a tetraparetic status (Frankel D). The patient was able to move the legs but could not walk without aid. Left hand was very weak (C6: 2/5 and C8: 3/5) with complete functional impairment.

**Fig. 1:**
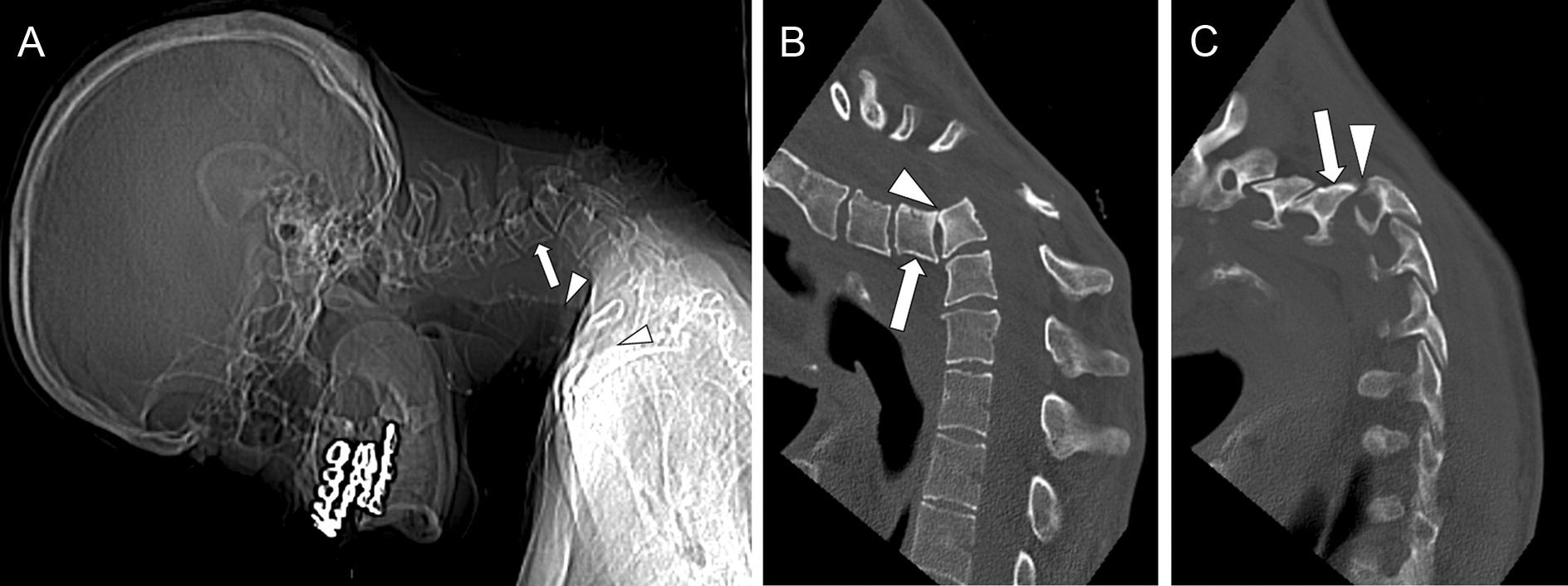
Initial computed tomography: scout view (**A**) showing the C4–C5 complete dislocation with spondyloptosis of C4 (arrow) and severe local kyphosis resulting in flexion of the head on the anterior chest wall with evident sinuosity of the respiratory tract (arrowheads, showing angulation of the trachea). Note the close proximity between the anterior aspect of the mandible and the sternum. Reformatted sagittal images on the midline (**B**) and at the level of the right facet joints (**C**) show complete dislocation at the C4–C5 level, with spondyloptosis of the C4 vertebral body anteriorly to C5 (arrow B) and complete anterior translation of the inferior C4 facet joint “locked” at the anterior aspect of the superior C5 facet (arrow in **C**). Sclerosis of the adjacent vertebral angles (arrowhead in **B**) and periarticular calcifications posteriorly (arrowhead in **C**) indicate the dislocation as established for several weeks

**Fig. 2:**
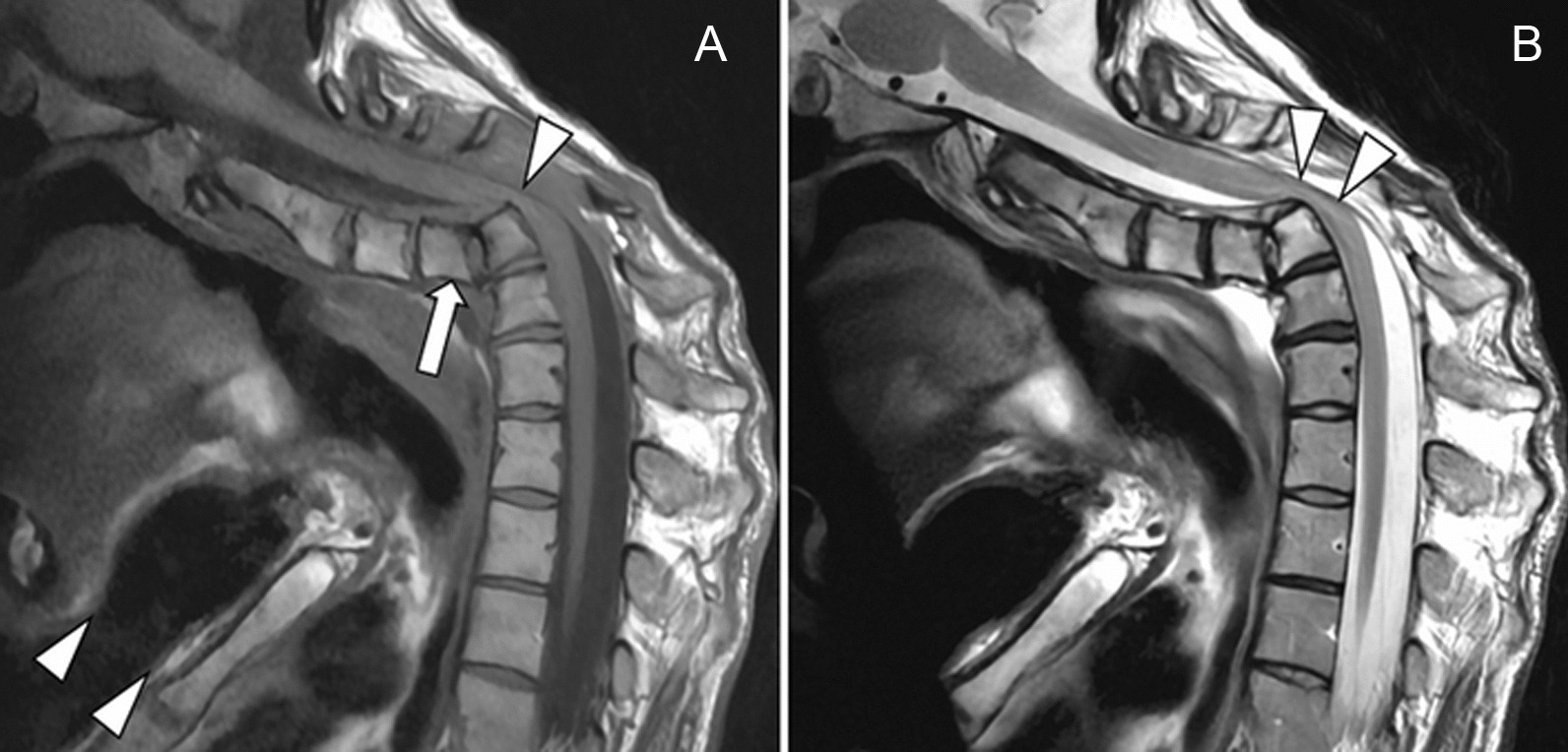
Initial magnetic resonance imaging: sagittal T1 (**A**) and T2 (**B**) weighted images showing the spondyloptosis of C4 (arrow) at the anterior aspect of the C5 body, and severe canal stenosis at the level of the posterosuperior angle of the C5 vertebral body (arrowhead in **A**). Note the close proximity between the chin and the sternum (less than 2 cm, arrowheads in **A**). Compressive myelopathy with high signal intensity of the spinal cord is evident on the T2 weighted image, involving the C4–C6 segment (arrowheads in **B**). The residual anteroposterior diameter of thecal sac was less than 5 mm

Radiographs, CT, and MRI showed a complete C4–C5 dislocation with spondyloptosis of the C4 vertebral body at the anterior aspect of C5 and a 90° kyphotic angulation between the upper and lower cervical spine segments (Fig. 1). The severe (100%) anterolisthesis of the C4 vertebral body and posterior arch was responsible for severe narrowing of the spinal canal with cord compression. MRI showed clear signal changes in the cord, consistent with compressive myelopathy (Fig. 2).

### Surgical and anesthesiologic management

A multidisciplinary discussion was held to elaborate on the treatment strategy.

Regarding anesthesia, the preoperative evaluation revealed a limited mouth opening (2 cm) and thyromental distance (2 cm), paralleling the severe cervicothoracic spine kyphosis. The landmarks for a possible tracheotomy were not accessible. A highly tricky airway access was expected, with a high risk of difficult ventilation and intubation. The anesthesia was also challenged by the continuous risk of neurological deterioration, neurogenic shock, and motor and sensitive deficit or respiratory failure during the perioperative and postoperative period.

Facing clinical and imaging findings, a stepwise surgical strategy was planned, consisting in an initial trial of closed reduction of the dislocation, followed by an open reduction and short-segment spine fixation in case of failure of the closed approach. This approach was planned after in-depth reflection and consideration of literature. The theoretical possibility of performing a C4-corpectomy and fixation in situ was not considered to be a reasonable surgical option because it would have fixed the head drop and kyphosis, compromising airways, and most importantly, limiting patient autonomy and head motion [12]. Indeed, it is well known, for example, in patients with spondyloarthritis, that fixation in severe kyphosis restricts the patient’s field of vision to the feet [13]. This was even more important in this particular patient due to his significant oculomotor limitation. The first mandatory step of the surgical procedure was a posterior approach to remove the facets, protect the vertebral arteries, have visual control over them, and avoid injuring them during the next surgical steps. If reduction could not be obtained during this posterior approach, an anterior approach would be necessary for reduction and anterior fixation, followed by a second posterior approach for strengthening the short-segment stabilization.

Two days after admission, a light continuous head traction (3 kg) was initiated using a halo. During this procedure, a local scalp anesthesia was performed with 2% Xylocaine, and oxygen was administered with goggles, with careful monitoring performed using a saturometer. As the procedure provided nonsignificant correction of the displacement and no neurological improvement, the weight was progressively increased to 6 kg. However, the follow-up X-ray showed only a subtle (2 mm) reduction of the displacement, confirming that this chronic (developing for almost 3 months) dislocation was not reducible.

Five days after admission, an open surgical reduction and stabilization procedure was performed in three steps. To be prepared to face the possible occurrence of a neurologic degradation and of cardiogenic shock, complete monitoring [electrocardiogram (ECG), arterial line, noninvasive blood pressure (NIBP)] was installed, and both a peripheral venous line and a central line were set up in case vasopressor injection was needed [14]. Due to the severe spine angulation and compromised airway access, an awake fibroscopic oral intubation was performed for the intraoperative period, using local anesthesia (Xylocaine 2% spray) and light sedation (remifentanil 0.1 mcg/kg/min), and after preoxygenation using a high-flow nasal oxygen therapy (Optiflow) with capnography. Light sedation allowed for contact with the patient to be maintained while providing comfortable intubation conditions.

**Fig. 3:**
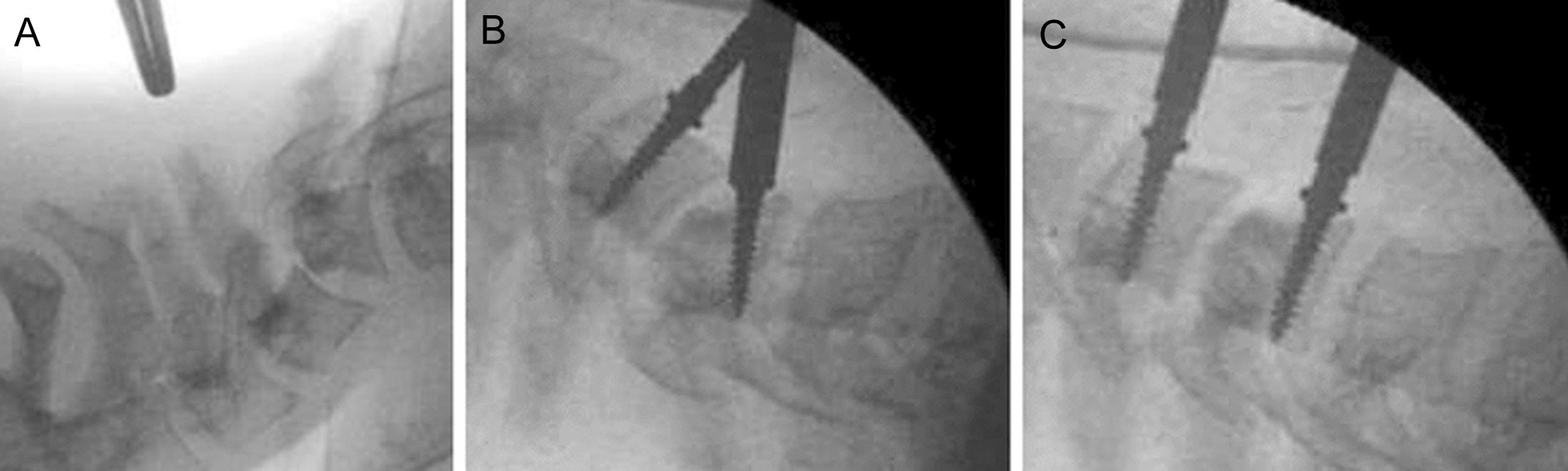
Lateral fluoroscopic view during the first posterior surgical approach (**A**) showing only very limited reduction of the C4 displacement. Lateral views during the subsequent anterior approach (**B**, **C**) show placement of screws within the C4 and C5 vertebral bodies (arrows in **B**) and correction of the displacement with repositioning of the C4 vertebral body (**C**), allowing for perfect realignment of the spine

The patient was first placed in the prone position with an axial head traction (4 kg). The dislocated C4–C5 facets were approached and the upper half of the C5 facets were removed under microscope, taking great care not to injure the vertebral arteries that were located immediately adjacent to the facets. This posterior approach failed to reduce the displacement and dislocation because of the retracted anterior longitudinal ligament (Fig. 3A). The patient was then placed in the supine position, again with a light head traction (2 kg). After a classic anterior cervicotomy, the anterior longitudinal ligament and longus coli muscle were transected just below the C4 vertebral body. Distraction pins were placed in C4 and C5 bodies and progressive and successful anterior and posterior reduction was obtained (Fig. 3B, C). To stabilize this C4–C5 segment, the disc remnants were removed, a 4 mm polyetheretherketone (PEEK) cage was inserted between the endplates, and an anterior stabilization plate was fixed to the vertebral bodies.

**Fig. 4:**
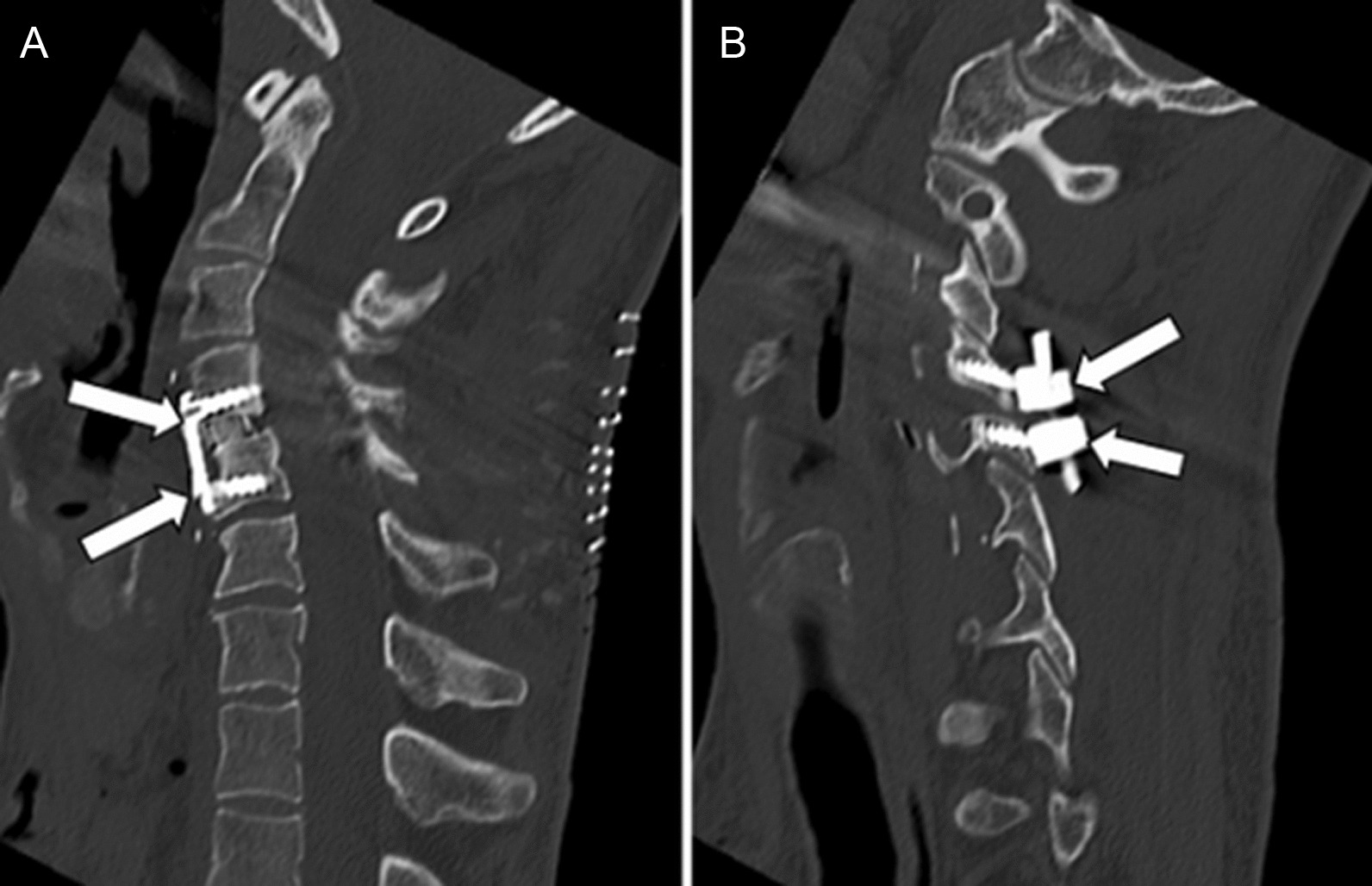
Postoperative computed tomography: reformatted sagittal images (at the same levels of preoperative images illustrated in Fig. 1B, C) showing the realignment of the spine, the anterior fixation using an intervertebral cage and an anterior plate in C4–C5 (arrows in **A**), and the posterior fixation using facet screws (arrows in **B**)

The patient was then repositioned in the prone position. The posterior operative field was reopened, C4 and C5 facet screws were placed, and facets were fixed in compression to achieve optimal stabilization (Fig. 4).

Short (rather than extensive) segment fixation was chosen to allow the patient sufficient head and neck movement and vision. Extensive fixation would have compromised his vision, as discussed above. 

**Fig. 5:**
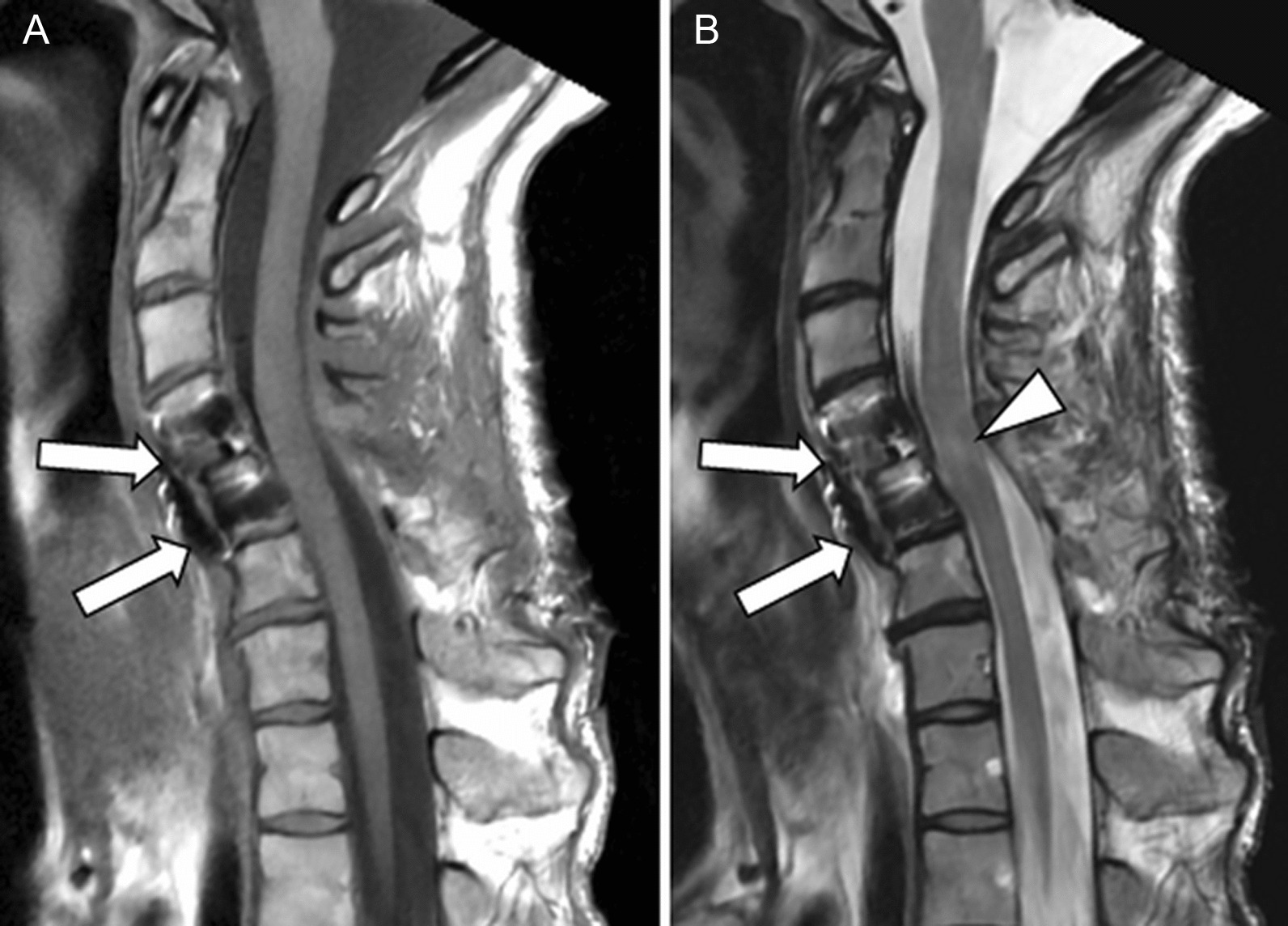
Postoperative magnetic resonance imaging: sagittal T1 (**A**) and T2 (**B**) weighted images showing realignment of the spine, return to normal dimensions of the spinal canal, and the presence of stabilization material (arrows). Note the presence of subtle residual myelopathy on the T2 image (arrowhead in **B**)

Throughout the surgical procedure, the anesthetic and surgical teams carefully managed the patient’s positioning, taking into account the stability of the spine and the risk of hemodynamic changes associated with changes in position. Between each step, the patient was awakened to assess the maintenance of motor function in the limbs. At the end of the operation and after ensuring that the motor function of the four limbs was preserved, the patient was transferred intubated to the intensive care unit for a more comfortable awakening, given the duration of the operation (10 hours) and the risk of extubation. Postoperative CT and MRI confirmed the successful decompression of the spinal canal, with complete reduction of the dislocation and stabilization of the C4–C5 level. Fluid-sensitive MRI sequences revealed a subtle (< 1 cm) residual signal abnormality in the spinal cord (Fig. 5).

Despite this, the patient made a full neurological recovery a few days after surgery. He was discharged home 1 week after surgery. The patient has had regular clinical and imaging follow-up for more than 2 years, and is asymptomatic. The sensorimotor examination at the last follow-up shows complete neurological recovery: left and right upper extremity examination shows a motor score of 5/5 and a sensory score of 2/2 for all C6, C7, C8, and T1 nerve roots. Upper and lower extremity examination shows normal reflexes. Last follow-up radiographs show no deterioration of the orthopedic hardware and adjacent disc levels.

## Discussion and conclusion

Cervical spine dislocation usually has an evident traumatic origin [2]. Rarely, post-traumatic cervical dislocation may be ignored at the early phase, and cases of “delayed” or “neglected” post-traumatic dislocation have been reported [5, 6]. An exceptional case of non-traumatic C4–C5 subluxation after cervical laminoplasty has been reported [15].

This case illustrates the observation of a severe non-traumatic occult (complete) C4–C5 dislocation in a patient with a predisposing condition. Indeed, the dislocation was observed after a recent history of evolving neck deformity and neurological deficit in a young man with immune myositis. On reflection, the dislocation may have been facilitated by physiotherapy sessions in a patient with severe muscle wasting and weakness associated with long-standing muscle atrophy.

In terms of clinical findings, the severe motor deficit indicated an urgent imaging study. The cervicothoracic deformity and neurological status suggested a challenging anesthesia and subsequent perioperative management. This case report highlights the importance of multidisciplinary coordination.

The imaging workup allowed for a thorough diagnosis and surgical planning. Radiographs and CT showed a complete dislocation with locked facets at the C4–C5 level. The displacement was particularly severe, as the C4 vertebral body had more than 100% anterolisthesis and a “fall” on the anterior aspect of the C5 body, described as anterior “spondyloptosis” of C4 [8, 16]. In contrast to previous observations combining spondyloptosis and traumatic separation of the posterior vertebral elements (spondylolysis), the present case was characterized by an intact posterior arch responsible for severe kyphosis and head drop. The term “kyphotic spondyloptosis” best describes this observation.

MRI is the method of choice to evaluate the severity of canal stenosis and detect spinal cord injury and potentially harmful associated disc herniation [17, 18]. Here, MRI confirmed the dislocation, ruled out disc herniation, showed severe cord compression, and established myelopathy. It illustrated the major deformity of the cervicothoracic junction compromising airway access.

The role of the anesthetist was cardinal throughout the therapeutic approach. The established spinal cord compression and myelopathy required careful management of intubation and mobilization of the patient, with constant attention to neuroprotection and monitoring of vital and neurological parameters.

Importantly, the irreducible severe airway deformity associated with the severe kyphosis made intubation difficult. This difficulty has been highlighted in other conditions with severe cervical-thoracic deformity [19]. Facing an unstable cervical spine and difficult airway access, awake fiberoptic bronchoscope intubation should be preferred to classical laryngoscopy [20, 21]. This guidance allowed for successful management of intubation in the current situation.

Spinal cord injury above the T6 level may be responsible for cardiogenic shock associated with autonomic dysreflexia, leading to hypotension and cardiac arrhythmias. To detect and be prepared for this theoretical risk of cardiogenic shock, appropriate monitoring was established during the induction phase of anesthesia (arterial line, ECG), as well as large peripheral venous and central lines. Particular attention was paid to all parameters during the perioperative position changes.

With this support, surgery was successfully performed. Initially, an attempt to reduce the dislocation by cranial traction was unsuccessful. The low success rate of traction reduction has been highlighted in dislocations that develop over several weeks, as fibrosis of the articular capsules and paraspinal ligaments fix the displacement. Facing complete spinal dislocation, different techniques have been reported, with variations in anterior or posterior approaches and number of operative steps [1, 9, 22]. Here, a three-stage open procedure allowed for reduction of the dislocation, decompression of the spinal cord, and combined anterior and posterior stabilization, resulting in a rapid and fortunately complete clinical and neurological recovery.

Corpectomy and fixation could have been considered as a therapeutic option, but they would have fixed the deformity. However, as reduction of the kyphosis was a priority given the severity of the deformity, reduction followed by fixation was the preferred option. As reduction was achieved during the surgical procedure, corpectomy was not needed.

We report the unusual observation of a complete non-traumatic cervical spine dislocation with severe fixed deformity. Multidisciplinary management allowed for successful management of a challenging spinal and neurological situation.

## Data Availability

Yes.

## Abbreviations

CT: Computed tomography
ECG: Electrocardiogram
MRI: Magnetic resonance imaging
NIBP: Noninvasive blood pressure
